# Enhancing the Photocatalytic Performance of WO_3_/AgBr Composites Through the Incorporation of Olive Waste-Derived Biochar Obtained Under Controlled Pyrolysis Conditions

**DOI:** 10.3390/ijms262110451

**Published:** 2025-10-28

**Authors:** M. Carmen Hidalgo, María D. Alcalá, José A. Navío, Francisca Romero-Sarria

**Affiliations:** 1 Instituto de Ciencia de Materiales de Sevilla (ICMS), CSIC—Universidad de Sevilla, Americo Vespucio 49, 41092 Sevilla, Spain; navio@us.es (J.A.N.); francisca@us.es (F.R.-S.); 2 Departamento de Química Inorgánica, Universidad de Sevilla, Profesor García González 1, 41012 Sevilla, Spain; mdalcala@us.es

**Keywords:** biochar, olive pruning, photocatalysis, WO_3_, AgBr

## Abstract

The integration of biochars into photocatalytic systems to increase their efficiency in the degradation of different pollutants in water has gained attention in recent years. However, systematic studies on optimizing biochar properties for photocatalysis remain limited. This work explores the incorporation of biochar from olive pruning (BCO), produced via CO_2_ pyrolysis at 800 °C, into WO_3_/AgBr photocatalysts for Rhodamine B degradation used as a model pollutant. Characterization of BCO reveals a hydrophilic, porous material (487 m^2^/g surface area) rich in mineral content (notably CaCO_3_). The study evaluates the effects of incorporation method (mechanical vs. in situ) and biochar content (1 and 10 wt. %) on photocatalytic performance. Comprehensive characterization of BCO and the resulting composites supports the observed activity trends. The findings highlight the potential of agricultural waste valorization for environmental remediation and offer insights into designing efficient biochar-based photocatalytic systems.

## 1. Introduction

Heterogeneous photocatalysis continues to be widely studied for a number of environmental applications, including the degradation of pollutants in water, gas or soil [1], disinfection [2], clean energy production such as hydrogen and hydrocarbons [3], or the green synthesis of chemicals of interest [4]. However, semiconductor-based photocatalysis using classical photocatalysts such as TiO_2_ and ZnO is still limited by insufficient efficiency mainly due to high carrier recombination of photogenerated carriers and poor absorption in the visible region. In general, the characteristics that a good photocatalyst material should have are: (i) low recombination of charge carriers, (ii) absorption of light over a wide range of wavelengths, including the visible, and (iii) good redox capability, which comes from appropriate band positions. In addition, since photocatalysis is a surface process, a high specific surface area with appropriate porosity is usually also advantageous [5,6].

One strategy to overcome high carrier recombination and to obtain appropriate band positions is the formation of heterojunctions by coupling two semiconductor photocatalysts. A heterojunction can be defined as the formation of an interface between two semiconductors with different band structures for band alignment. The goal in constructing a heterojunction is to increase the spatial separation of the electron–hole pairs and thereby increase their lifetime. In this way, more charge carriers can reach the surface of the photocatalyst and participate in the redox half-reactions of the photocatalytic process. Depending on the band positions of both semiconductors, band gaps and electron migration paths, different types of heterojunctions can be formed, the most common being the type II, the Z-scheme and the S-scheme [7,8,9,10].

Another strategy that has been studied lately to increase the efficiency of photocatalytic materials involves coupling or depositing them onto a carbonaceous material with high specific surface area. This carbonaceous material is often a biochar (BC) obtained from biomass residues, which gives it the added value of waste utilization in the context of the circular economy [11,12,13,14]. Biochars are produced by thermal treatment of organic matter (biomass) in the absence of oxygen (pyrolysis), and a great variety of biomass can be used as feedstock, including agricultural, forestry and food wastes. These materials exhibit interesting properties such as high specific surface areas and porosities, and can be tailored by controlling the operating conditions to obtain desired amount and type of functional groups on their surfaces, porosity, hydrophobicity or hydrophilicity, surface pH, etc.

Coupling photocatalysts to biochar can provide the materials with attractive properties in terms of photocatalytic efficiency. On the one hand, biochar can provide high specific surface area to the systems, increasing the potential adsorption of the pollutants. This in turn can enhance photocatalysis by improving the contact between the pollutants and the photocatalyst. On the other hand, since biochars are good conductors, the rapid recombination of photogenerated charges can be reduced by facilitating their spatial separation. And moreover, biochars can also act as photosensitizers, narrowing the band-gap of the systems [11,12,13,14]. It is foreseeable that all these characteristics will enhance the efficiency and photocatalytic performance of the materials.

In addition to the nature of the biomass, the pyrolysis conditions used to produce the biochar are of great importance for the final properties of the material. They determine not only product yields, but also the structure and functional groups of the biochar. At high pyrolysis temperatures, the specific surface area and pore volume increase (about 700–800 °C) due to the elimination of species and intermediates from the carbon surface. However, at higher pyrolysis temperatures, the internal structure breaks down and the surface area decreases [15]. Moreover, the degree of graphitization of the biochar increases with increasing temperature, and the amount of hydrophilic functional groups on the surface as well as the O/C and H/C ratios decrease. Therefore, the operating conditions must be carefully selected for each type of biomass in order to obtain the desired properties of the biochar for the intended application.

In the context of the aforementioned background and combining the two strategies described above to improve the photocatalytic efficiency of materials, in the present work we have prepared heterojunctions of two narrow-bandgap semiconductors WO_3_/AgBr and coupled them to different percentages of biochar, obtained from olive pruning waste by pyrolysis under previously optimized conditions. Olive branch pruning is a common agricultural waste in southern Spain and other Mediterranean regions where there are large extensions of olive groves.

Recently there has been great interest in the development of photocatalysts with visible light absorption, and among these materials one with interesting properties is WO_3_ [16]. This material is a narrow-band-gap semiconductor (Eg value around 2.8 eV) that absorbs in the visible region of the spectrum, is chemically and photochemically stable in acidic and oxidative conditions, is non-toxic, and is relatively inexpensive. However, by itself, it is a very limited photocatalyst due to its fast recombination of photogenerated charges and to its low reduction ability due to its positive conduction band potential [17,18]. To overcome these limitations, in a previous work [19] we prepared WO_3_/AgBr type II heterojunctions with different ratios between the two components with good activity results for rhodamine B and caffeine degradation. However, these systems had low specific surface areas (<10 m^2^/g). Therefore, in the present study, we prepared WO_3_/AgBr by a different method and incorporated different percentages of biochar by two different routes. For the preparation of WO_3_, instead of the conventional hydrothermal method, a microwave-assisted hydrothermal method was used, which uses relatively low reaction temperatures, shorter reaction times, and allows obtaining more homogeneous and reproducible materials with controlled particle size [20].

In the design of photocatalyst/biochar coupled materials, the preparation route is also of great importance and can have a direct influence on the photocatalytic behavior of the systems [21]. In this work, we have investigated two of them: mechanical–physical mixing and in situ incorporation of the biochar into the synthesis medium of the WO_3_/AgBr system. Furthermore, as mentioned above, the operational conditions of biomass pyrolysis are crucial for modelling the properties of the final biochar. In our study, the pyrolysis conditions were chosen to obtain a high specific surface area and hydrophilic biochar.

In the present work, the preparation of WO_3_/AgBr/Biochar composites is addressed for the first time, using a biochar obtained by pyrolysis under optimized conditions of olive branch pruning. The importance of the conditions of biochar preparation and the method of its introduction into the photocatalytic system is emphasized. The obtained results are promising for the development of efficient materials for photocatalytic applications.

## 2. Results and Discussion

### 2.1. Characterization of the Materials

#### 2.1.1. Biochar

The characteristics of a biochar are strongly dependent on the pyrolysis conditions under which it is produced and on the nature and composition of the starting biomass; in our case, the biochar (BCO) was produced from olive pruning waste by pyrolysis at 800 °C in CO_2_ flow as described in the experimental section. Prior to this, biochar was prepared under different temperature conditions and by varying the carrier gas. It was observed that the specific surface area of the solids increased with the temperature of treatment under CO_2_ flow; therefore, a working temperature of 800 °C was selected. Moreover, pyrolysis under an Ar flow at low temperatures resulted in a strongly hydrophobic material, which hindered coupling with the photocatalytic system and its use in aqueous media, whereas pyrolysis under a CO_2_ flow at 800 °C produced biochar with hydrophilic characteristics. The hydrophilic/hydrophobic properties of the obtained solids were assessed using the water drop penetration time (WDPT) test [22] and the pyrolysis conditions that yielded hydrophobic materials were discarded.

Considering the obtained results and the application of these materials, the selected pyrolysis conditions were 800 °C under a flow of CO_2_. The use of CO_2_ in thermochemical processes is receiving considerable attention, both for the properties it imparts to the resulting products and for its potential contribution to CO_2_ mitigation. Thus, the use of CO_2_ as pyrolysis gas is a very interesting aspect due to both, the properties of biochar induced by this gas (hydrophilic character and high specific surface area) and for its contribution to CO_2_ mitigation. In this context, the use of CO_2_ in gasifiers has progressed from laboratory-scale to pilot-scale applications for biomass gasification, and large-scale production of biochar by pyrolysis is not only feasible, but also economically advantageous [23,24].

The biochar obtained under these conditions has a hydrophilic character and its elemental analysis indicates an overall carbon content of 53 wt. % (Table 1). On the other hand, the proximate analysis of the raw biomass (olive tree pruning) produces the relationship shown in Table 1. Assuming a yield-to-solid of 19 wt. % (as determined experimentally), the obtained biochar has a mineral content of 23 wt. % (oxides and carbonates), and the rest would correspond to the amorphous carbonaceous compounds that constitute the biochar.

To find out what other elements were present in the BCO, we carried out an XRF analysis, since the elemental composition of biochars can be very variable due to their inherent nature. The results are presented in Table 2, where the elements present in the sample (except C, H, N and O) are listed. As can be seen, the main inorganic elements present are Ca and K, significantly above the rest, although elements such as Si, P and Mg can also be found.

**Figure 1:**
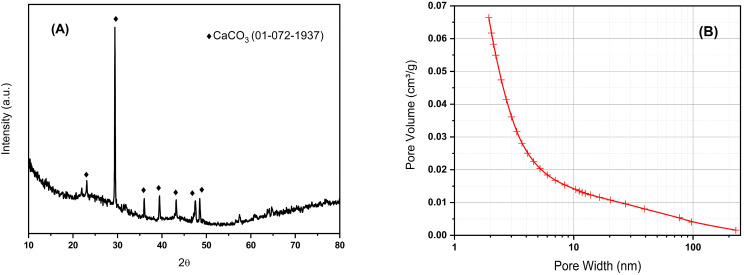
XRD pattern (**A**) and pore size distribution (**B**) for biochar obtained by pyrolysis of olive tree prunings at 800 °C in CO_2_.

The X-ray diffraction pattern of BCO is shown in Figure 1A. It can be seen the amorphous structure of biochar in addition to a certain amount of well-defined crystalline phase identified as CaCO_3_ (PDF card #01-072-1937); this fact is consistent with the XRF results where a high content of calcium was observed. No diffraction peaks of other inorganic compounds are observed. The broad peak between 18–28 degrees 2Ɵ observed due to carbon is related to the degree of orientation of the aromatic lamina formed during pyrolysis, where the sharper this peak is, the higher the degree of condensation of the aromatic rings [25]. The specific surface area of the BCO was 487 m^2^/g, with a total pore volume of 0.25 cm^3^/g, and estimated micropore and mesopore volumes 0.17 and 0.06 cm^3^/g, respectively. The pore volume distribution was mostly concentrated in the range of less than 10 nm pore width, as can be seen in Figure 1B. The obtained biochar not only exhibits a specific surface area comparable to that of catalysts used in conventional catalytic processes, but it is also hydrophilic, making it suitable for treatments in aqueous media. Its hydrophilic nature may be attributed to the formation of oxygen-containing functional groups on the biochar surface when CO_2_ is used as the pyrolysis gas and the evaporation of aliphatic compounds at high temperatures.

**Figure 2:**
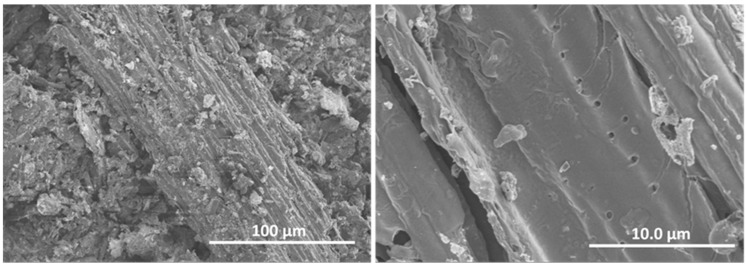
Representative SEM images of biochar: general view (**left image**) and magnified view (**right image**).

The morphology of the biochar BCO was studied by SEM and representative images are shown in Figure 2. It can be seen that BCO consists of rather heterogeneous particles of relatively large size, approximately 200–500 microns, elongated in shape and fibrous in appearance. The higher magnification image (Figure 2 right) shows the porous structure of the particle surface.

**Figure 3:**
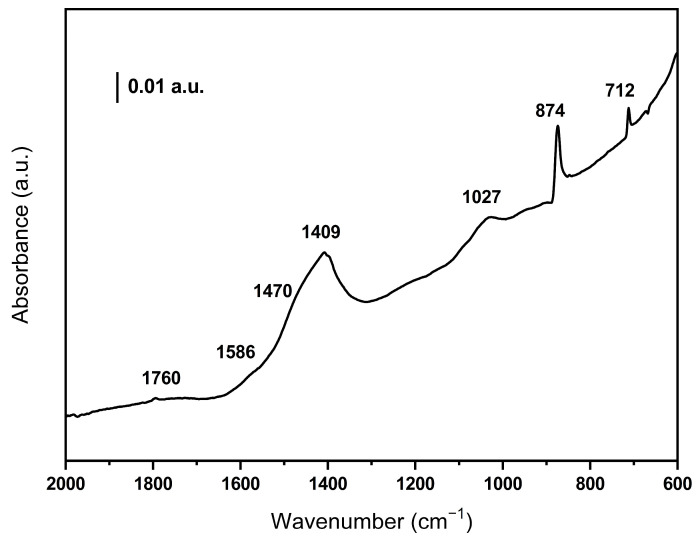
DRIFT spectrum of the olive waste-derived biochar.

Figure 3 shows the IR spectrum of the BCO sample. No bands are observed in the high wavenumber region where the O-H and C-H vibrations are detected, indicating that the biomass has dehydrated and decomposed after the pyrolysis treatment. Weak bands can be detected at 1760 and 1027 cm^−1^ which are due to C=O and C-O groups, respectively. It should be noted that the bands around 1400 cm^−1^ confirm the presence of carbonate species in the sample. In addition, the weak band at 1586 together with those observed at 712 and 874 cm^−1^ indicate the presence of aromatics (the 874 band overlaps with the π-vibration of the carbonate species) [26].

As mentioned above, the aim of introducing biochar into the photocatalyst is to obtain systems with a higher specific surface area, to increase the lifetime of the charge carriers and to reduce the band gap [11,12,13,14]. However, in addition, the presence of CaCO_3_ in our biochar should be taken into account when analyzing the photocatalytic activity data of the WO_3_/AgBr/BCO systems, as it has been reported that CaCO_3_ can also have photocatalytic activity by itself due to the possible formation of ·CO_3_^−^ under illumination in the presence of suitable photocatalysts [27,28]. Unlike other radicals produced in the photocatalytic processes, such as ·OH, ·CO_3_^−^ radicals are more selective and can reach a higher steady state concentration in solution [29].

An important property sought in biochar, in addition to a high specific surface area, is that it should have a hydrophilic character and high wettability to facilitate adsorption and interaction with the molecules in the aqueous medium to be degraded. The hydrophilic character of biochar depends on the content of hydrophilic groups on the surface and the presence of inorganic species [30]. Hydrophilic groups consist mainly of oxygenated species, the formation of which can be promoted by the use of carbon dioxide (CO_2_) as a pyrolysis gas. In our case, this is confirmed by the presence of infrared bands associated with C-O bonds (Figure 3). In addition, materials obtained in the presence of CO_2_ have a high specific surface area. On the other hand, it is known that the presence of alkali and alkaline earth elements in the initial biomass hinders the aromatic rings condensation, thus reducing the hydrophobic character of the biochar [31]. As a result, the obtained biochar is a hydrophilic material with a high specific surface area.

#### 2.1.2. Photocatalysts/Biochar Systems

**Figure 4:**
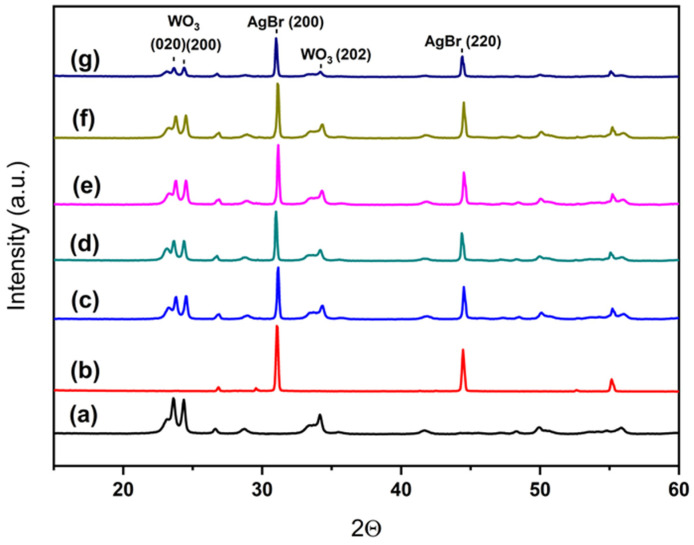
XRD patterns for the different systems: WO_3_ (a), AgBr (b), WO_3_/AgBr (c), WO_3_/AgBr MM 1% BCO (d), WO_3_/AgBr MM 10% BCO (e), WO_3_/AgBr DP 1% BCO (f), and WO_3_/AgBr DP 10% BCO (g).

X-ray diffraction patterns for all samples are depicted in Figure 4. As it can be seen, pure WO_3_ presents a single monoclinic phase (PDF card #00-043-1035) and AgBr presents well defined cubic AgBr peaks (PDF card #00-006-0438). For the composite systems WO_3_/AgBr/BCO, the diffraction peaks of both parent compounds are observed in all samples, while no peaks corresponding to biochar (CaCO_3_) are detected. This absence is attributed to its low weight content in the samples and the relatively low amount of CaCO_3_ in the BCO. Although CaCO_3_ is the only crystalline phase present in the biochar, its content does not exceed 23 wt. %, as previously stated.

In our previous study [19], it was found that the WO_3_/AgBr system showed enhanced photocatalytic activity due to the synergistic effect between the two components as a result of the WO_3_/AgBr heterojunction. However, due to the very low specific surface area of AgBr (<1 m^2^/g), these systems had a low final surface area. Therefore, one of the motivations for introducing biochar into these systems was to provide them with a higher specific surface area. The SBET values for all samples are shown in Table 3. It can be seen how the introduction of biochar increases the BET surface area of the system. In the case of the samples with 1% BCO, the increase depends on the method used for carbon addition and the surface area is higher (about 18 m^2^/g) for the sample where the BCO is introduced in situ during the preparation of the photocatalyst. For the samples with 10% BCO, the specific surface area is further increased to around 37–38 m^2^/g, regardless of the method used for biochar introduction.

It is known that the introduction of biochar can reduce the band gap of a photocatalyst system. This reduction may be due to several factors conferred by the biochar, such as photosensitization, the introduction of intermediate energy states in the forbidden band of the photocatalyst, and the introduction of local trapped states [12,32,33,34]. However, our starting photocatalyst, the WO_3_/AgBr heterostructure, already has a rather low bandgap (2.65 eV) as it is composed of two narrow-bandgap semiconductors, and the introduction of BCO into the system does not affect the bandgap value of the system. Table 3 lists the bandgap values of all samples calculated from the UV-Vis absorption spectra using the Kubelka-Munk function (F(R∞)), which is proportional to the absorption. It can be seen that the systems with BCO have band gap values equal to or slightly higher than the photocatalyst without biochar.

**Figure 5:**
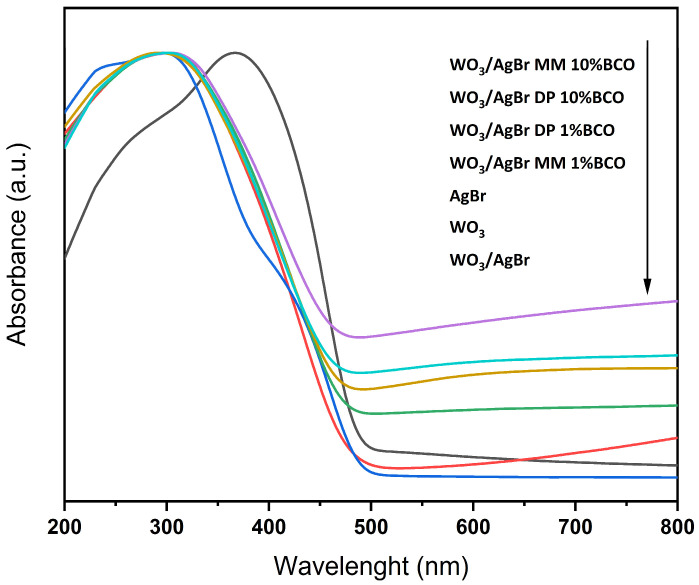
UV-Vis absorbance spectra for the indicated catalysts. The arrow indicates from top to bottom.

Figure 5 shows the UV-Vis absorption spectra of the different samples. It can be seen that although the absorption edge does not vary significantly between the samples with BCO, as reflected in the calculation of the corresponding band gaps, the absorption over the visible range increases with the BCO content. As expected, samples with higher biochar content are darker and have higher absorption throughout the visible.

**Figure 6:**
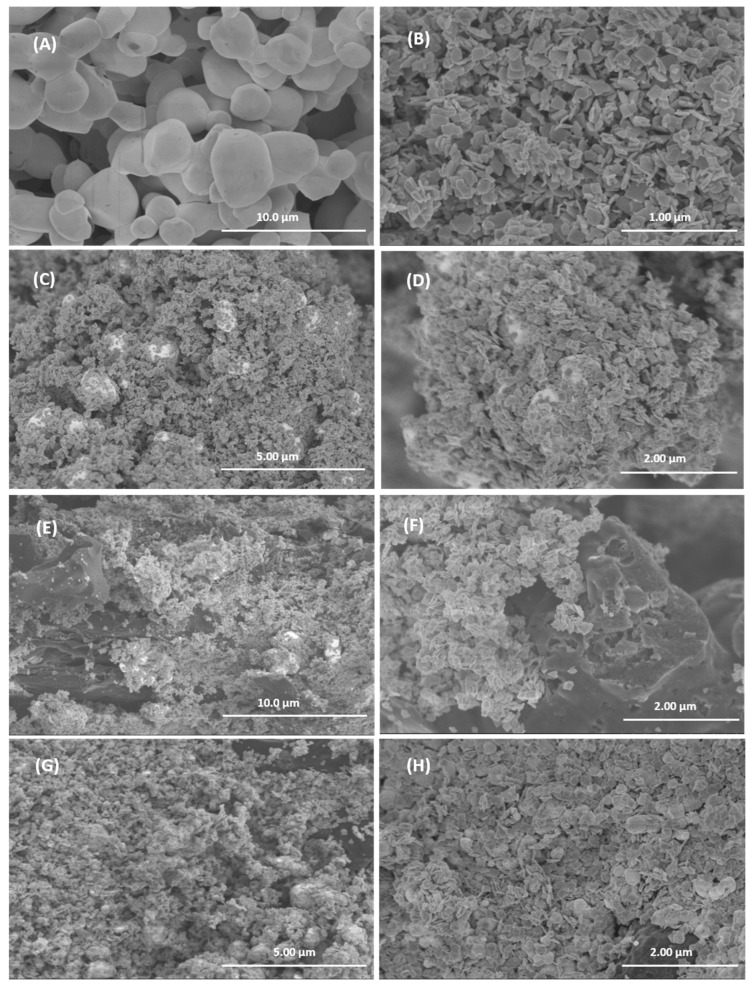
Representative SEM images for AgBr (**A**), WO_3_ (**B**), WO_3_/AgBr (**C**,**D**), WO_3_/AgBr MM 10% BCO (**E**,**F**), WO_3_/AgBr DP 10% BCO (**G**,**H**).

The morphology of the different samples was studied by SEM and representative images are shown in Figure 6. The individual compounds are shown in Figure 6A (AgBr) and 6B (WO_3_). The AgBr sample consists of interconnected quasi-spherical particles of approximately 1 to 3 microns. WO_3_ consists of platelet-shaped particles with rounded edges and a side length of about 100 nm. In the WO_3_/AgBr system (Figure 6C,D), the WO_3_ particles are located on the surface of the AgBr spherical particles (white spheres in the images), in a morphology similar to that found in previous work for these materials [19]. Figure 6 also shows representative images of the WO_3_/AgBr systems after the addition of 10% *w*/*w* BCO biochar by mechanical mixing (Figure 6E,F) and by in situ addition (Figure 6G,H). As can be seen, in both methods the WO_3_/AgBr particles are placed covering the surface of the biochar particles, although it is noticeable that in the sample with the in situ addition (Figure 6G,H) the coating of the BCO is more effective than in the sample with addition by physical mixing (Figure 6E,F). In the former almost the entire surface of the biochar is coated by the WO_3_/AgBr particles, whereas in the mechanical addition significant areas of uncovered biochar particles are observed. Thus, it can be concluded that the in situ method of biochar introduction produces is more effective coatings and a more homogeneous morphology than the mechanical mixing.

**Figure 7:**
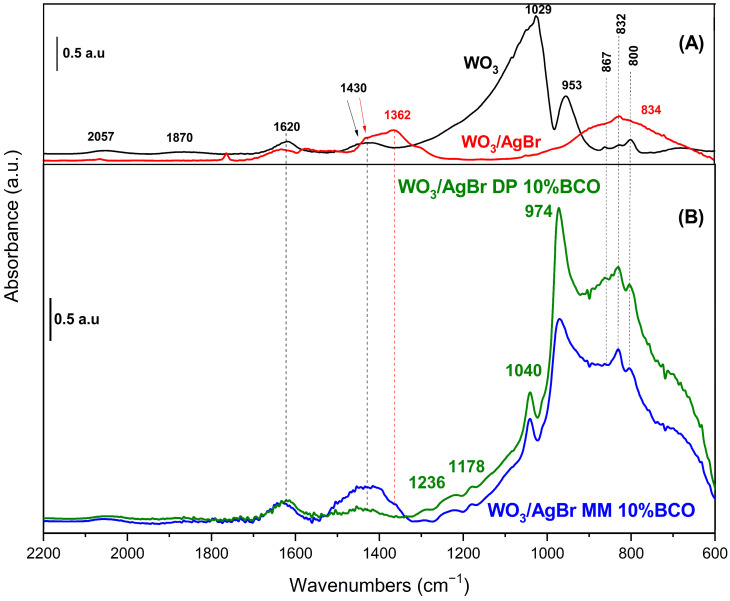
DRIFT spectra of (**A**) WO_3_ (black line) and WO_3_/AgBr (red line); and (**B**) WO_3_/AgBr DP 10% BCO (green line) and WO_3_/AgBr MM 10% BCO (blue line).

Figure 7 shows the DRIFT spectra of the samples. In the WO_3_ spectrum (black trace), bands at 800, 832 and 867 cm^−1^ attributed to skeletal vibrations, W-O-W and O-W-O of the oxide are observed [35,36]. The bands at 953 and 1029 cm^−1^ are due to W-O vibrations with different bond order. The WO_6_ octahedra, which constitute the monoclinic structure of WO_3_, exhibit distortion, manifesting two distinct values for the W-O bond distances (four short and two long bonds). Accordingly, the bands observed at 953 and 1029 cm^−1^ can be ascribed to both types of W-O bonds, with bond order around 1 and 2, respectively [37]. In addition, groups with a higher bond order are mainly found on the surface of the solid, which could explain the high intensity of the band at 1029 cm^−1^ in the spectrum of pure WO_3_. The first overtones of these bands appear at 1870 and 2057 cm^−1^ and those observed at 1620 and 1430 cm^−1^ are assigned to the bending mode of adsorbed water molecules and to W-O-H species, respectively [36].

When AgBr is coupled to WO_3_ (red line in Figure 7), the DRIFT spectrum is strongly modified. Typical bands of AgBr appear around 650 and 920 cm^−1^ [38], the first one in the dark region of the spectrum and the other one overlapping with the broad band centred at 834 cm^−1^. The bands at 1362 cm^−1^ and 800 cm^−1^, suggest the presence of nitrate impurities, which is not unreasonable considering the AgBr preparation method employed [39]. In addition to these bands, the most significant difference in the spectrum of WO_3_/AgBr (compared to pure WO_3_) concerns the W-O bonds, with a strong decrease in the band at 953 cm^−1^ and the complete disappearance of that at 1029 cm^−1^. The W-OH species (1430 cm^−1^) remain unchanged. These observations suggest that W-O bonds play a crucial role in the WO_3_/AgBr coupling, probably through interactions involving “O” species, which would explain the observed modifications.

**Figure 8:**
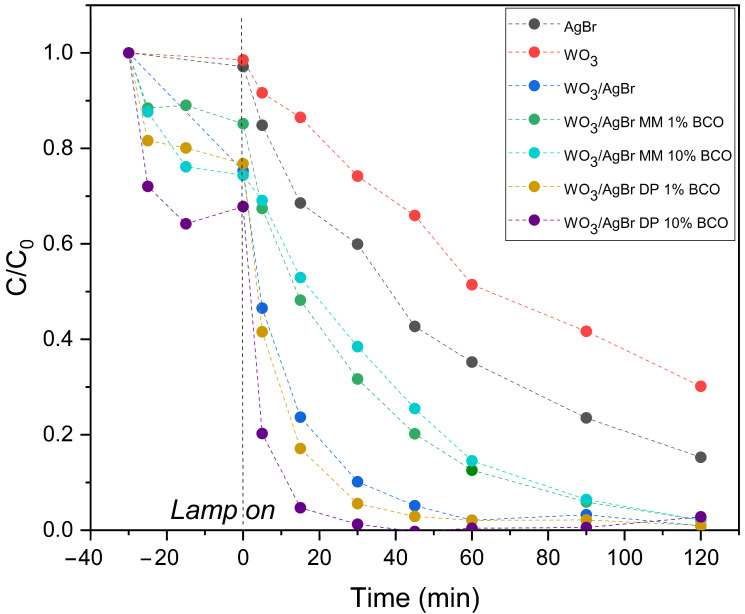

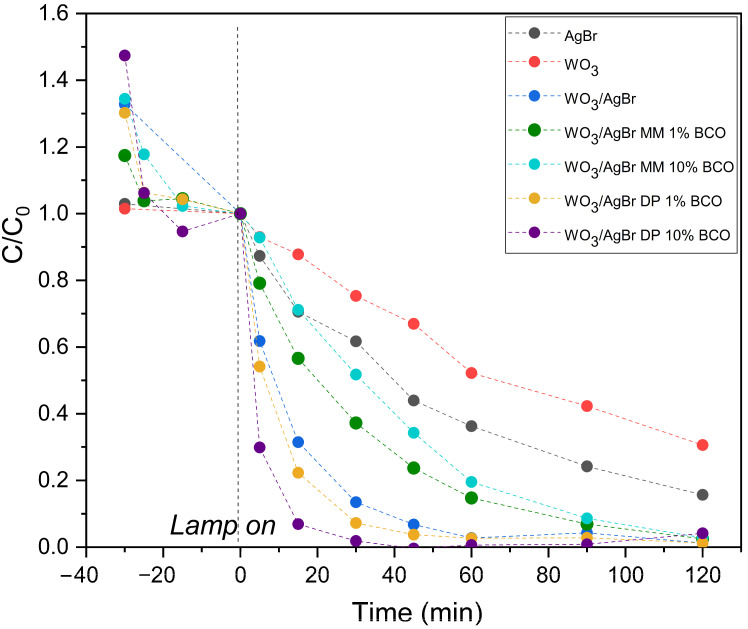
RhB degradation profiles over the indicated photocatalysts (**top graph**) and normalized to RhB concentration after adsorption equilibrium (**bottom graph**).

After the incorporation of BCO into the WO_3_/AgBr system (via both MM and DP methods) the presence of new bands at 974 and 1040 cm^−1^ is evident in the spectra (blue and green lines in Figure 7). The former is observed in the typical W-O bonds region although with a shift to higher wavenumbers in comparison to unmodified WO_3_. The observation of this band (missing in the WO_3_/AgBr material) can be elucidated by considering the affinity of Ag^+^ for different ligands. Ma’s calculations [40] showed that the affinity of Ag^+^ for oxygen-containing ligands is significantly lower than for aromatic compounds, and increases with the number of rings in the compound. Therefore, it can be hypothesized that in the prepared composites, Ag^+^ ions interact preferentially with BCO, releasing ‘O’ sites from the oxide and allowing the W-O bonds to be observed. Moreover, the blue shift in this band (now at 974 cm^−1^), which indicates a strengthening of this bond, can be related to the presence of a band at 1040 cm^−1^, indicative of the formation of a W-C bond (whose attribution is corroborated by the presence of weak bands at 1178 and 1236 cm^−1^) [41]. The perturbation of W-O bonds by the presence of W-C interactions has been previously reported in the literature [42,43]. The observations presented so far apply to both composites, obtained by MM and by DP methods. The main differences between the spectra of both composites are observed in the 1000–600 cm^−1^ region. The relative intensity of bands due to W-O bonds is much higher in the spectrum of the WO_3_/AgBr DP 10% BCO composite. This suggests a stronger interaction with silver, certainly induced by the preparation method. The higher content of nitrate impurities observed in the MM composite (band at 1362 cm^−1^) may reduce Ag-BCO interactions in this sample, reducing the intensity of bands in the W-O bond region. The differences in the rhodamine B adsorption and photodegradation observed for these samples (see Figure 8 and discussion) could be related to these facts.

### 2.2. Photocatalytic Runs

Rhodamine B (RhB) is a cationic xanthene dye, commonly used as a colorant in many applications, such as paint, textiles, paper, leather industries, etc., which can lead to serious harm to wildlife and has been confirmed as mutagenic and carcinogenic to humans [44,45]. In this work, RhB has been chosen as model pollutant to evaluate the photocatalytic performance of the different samples. RhB is a xanthene dye with the molecular formula C_28_H_31_ClN_2_O_3_. Its structure includes a xanthene core, two diethylamino groups, a carboxyphenyl substituent, and a chloride ion. The photocatalytic degradation pathway is well studied [46] and involves the breakdown of the chromophoric structure by reactive oxygen species (ROS) generated during the photocatalytic process, by cleavage of the xanthene ring and amino groups. The resulting intermediates are further oxidized to CO_2_ and H_2_O, leading to complete mineralization.

Figure 8 (top graph) shows the degradation profiles of RhB with illumination time over the different photocatalyst systems: WO_3_, AgBr, WO_3_/AgBr and WO_3_/AgBr/BCO with the two percentages of BCO and the two mixing methods. It is important here to separate the adsorption processes from the photocatalytic degradation processes, therefore the tests are performed with a first half hour in darkness, where several points are also taken to measure the dye concentration, that accounts for the adsorption capacity of each sample, which occurs mainly in the first minutes of contact between the catalyst and the RhB solution. The highest RhB adsorption occurs on the WO_3_/AgBr DP 10%BCO sample (about 30% of RhB). This sample has a higher RhB adsorption capacity than the WO_3_/AgBr MM 10%BCO sample, although both have the same specific surface area (37–38 m^2^/g). This improved adsorption capacity could be related to the stronger interaction Ag-BCO observed by DRIFTS analysis. The reductive character of the “C” could induce a higher surface electronic density, explaining the good adsorption of a cationic dye in this sample.

On the other hand, the degradation rate of RhB once the lamp is turned on is appreciably higher for the samples with BCO prepared by DP, especially for the higher percentage of BCO (WO_3_/AgBr DP 10%BCO). For the samples prepared by mechanical mixing (MM), the degradation rate of RhB not only does not increase with respect to the WO_3_/AgBr sample (without BCO), but decreases. As observed by SEM analysis, mechanical mixing (MM) does not provide good dispersion of the photoactive material on the biochar surface, which could limit the interaction between contaminant molecules adsorbed on the biochar and photocatalyst particles that are not in close proximity, thereby limiting the possibility for the ROS generated on the photocatalyst to reach the rhodamine molecules for their degradation. Furthermore, due to the nature of the mixing method, intimate contact between the photocatalyst and biochar particles is not achieved, thus hindering the role of biochar as a facilitator for the separation of photogenerated charges in the photocatalyst.

To better visualize the photocatalytic degradation slopes of the samples prepared by DP, the dye disappearance profiles have been normalized at zero illumination time, i.e., after adsorption equilibrium has been reached and at the moment the lamp is turned on, and these profiles are shown in the lower graph of Figure 8.

The kinetic constants (k, min^−1^) were calculated from the linear fit of ln(C/C_0_) versus time during the first 30 min of illumination. All fits show good agreement with first-order kinetics (R^2^ > 0.95). The results are presented in Table 4. As can be seen, the kinetic constant for the WO_3_/AgBr DP 10%BCO sample is more than twice that for the sample without biochar addition, confirming a significant improvement in reaction rate.

Thus, it can be observed that there is a synergistic effect between BCO and photocatalysts when BCO is introduced in situ during the preparation of WO_3_/AgBr systems, which could be justified not only by a higher specific surface area, but also by a better mobility of the photogenerated electrons and, therefore, a longer lifetime of the e^−^-h^+^ pairs, as reported in the literature for other systems with biochars [11,12,14]. In addition, biochars have been reported to exhibit significant redox activity due to their surface functional groups, graphitized and semi-quinonic structures, and conjugated π–electron systems. This could also allow biochar to actively participate in redox reactions, either by donating or accepting electrons. The so-called persistent free radicals (PFRs) in biochar could also catalyze the formation of ROS, thereby facilitating the photocatalytic process [47]. Likewise, the participation of the calcium carbonate present in the biochar through the generation of ·CO_3_^−^ radicals by the electrons generated by the illumination of the WO_3_/AgBr photocatalyst cannot be excluded [26,27,48].

As preliminary assessment of the catalyst stability, reusability tests were performed using the sample that exhibited the highest activity, i.e., WO_3_/AgBr DP 10% BCO, over four consecutive cycles of activity. After each cycle, the catalyst was recovered, dried overnight at 90 °C, and reused with a fresh 10 ppm RhB solution. The degradation curves and the corresponding kinetic constants (k, min^−1^) are provided in the Supplementary Information (Figure S1 and Table S1, respectively). No significant decrease in the degradation rate was observed during the first four cycles.

In summary, the incorporation of BCO into the WO_3_/AgBr systems during the synthesis process (DP) leads to a higher interaction between the biochar and the photocatalyst, as found by SEM and DRIFT analyses of these systems, which consequently makes them behave in a more efficient way in the photocatalytic degradation of RhB. This makes these systems promising materials for their use as efficient photocatalysts, which also provides a way to valorize an agroforestry waste in the context of the circular economy. The results obtained here could be extrapolated to other systems with other photocatalysts.

## 3. Materials and Methods

Biochar: The olive branches collected from the field (agricultural exploitation in Malaga, Spain, Hojiblanca type) were first manually cut and then processed using a knife mill (IKA-WERKAE, M20, Staufen, Germany). Subsequently, in order to obtain smaller particles, the sample was subjected to a second grinding process, this time using a ball mill (RESTCH, PM200, Haan, Germany) at 500 rpm for 20 min. A stainless steel jar (volume, 125 mL) with 9 stainless steel balls (diameter, 20 mm) was employed.

Then, the biomass was pyrolyzed in a vertical pyrolysis device (homemade), consisting of a quartz tube with a porous plate in the middle, placed inside a furnace that can be heated up to 1000 °C, and coupled to a set of liquid condensers. In a typical experience, about 10 g of biomass was treated at 800 °C and maintained 1 h at this temperature (heating rate of 10 °C/min) under a CO_2_ flow of 200 mL/min. The so obtained biochar was called BCO. These pyrolysis conditions were chosen because they lead to a biochar with moderately high specific surface area and hydrophilic character.

Photocatalyst preparation: The preparation of the WO_3_/AgBr catalyst was carried out according to the procedure described in [19], except that the WO_3_ was prepared by microwave-assisted hydrothermal method. Briefly, a solution of NaWO_3_ 99% (Sigma-Aldrich, Schnelldorf, Germany) in water was prepared and concentrated HCl 37% (Honeywell Fluka, Seelze, Germany) was added dropwise until a yellow precipitate was formed and left under magnetic stirring for 30 min. This precipitate was then transferred to a Teflon vessel and heated under microwave irradiation using a microwave reactor (Milestone, ETHOS One, Sorisole, Italy) at 100 °C with a power of 400 W for 2 h. The solid was recovered and washed several times with distilled water, dried at 90 °C overnight and calcined at 400 °C for 4 h at a heating rate of 5 °C min^−1^.

The WO_3_/AgBr-coupled material was then obtained by a simple in situ precipitation-deposition method with a WO_3_:AgBr molar ratio of 1:1 (optimum ratio according to [19]). A suspension of the above obtained WO_3_ containing the estimated amount of AgNO_3_ (for a WO_3_:AgBr molar ratio of 1:1) was prepared under constant magnetic stirring for 1 h in the dark. Then, a KBr solution was added drop by drop to form AgBr and stirred for 24 h at room temperature. Afterward, the solid was filtered, dried at 100 °C overnight and ground with a mortar.

Photocatalysts/Biochar: The addition of biochar to the WO_3_/AgBr photocatalysts was carried out by two different methods and at two percentages: 1% and 10% total weight with respect to WO_3_.

The first method consisted of a simple mechanical-physical mixing of the photocatalyst with the biochar by placing the appropriate amounts of the two components for percentages of 1 and 10 wt. % biochar in an agate mortar and mixing the two materials for a few minutes. These samples were labeled with the acronym MM and the percentage of biochar.

In the second method, the biochar was introduced during the synthesis of the photocatalyst, where the appropriate amount of biochar was added to the AgNO_3_ solution in suspension with the WO_3_ for 30 min before adding the KBr. Then the rest of the synthesis was performed as described in the previous section. The samples thus obtained were referred to as DP plus the percentage of biochar.

Characterization: Crystalline phase composition and degree of crystallinity of the samples were estimated by X-ray diffraction (XRD), on a PANalytical X’PERT PRO diffractometer and X-ray fluorescence was performed on a wavelength-dispersive X-ray fluorescence spectrometer PANalytical ZETIUM (Malvern Panalytical B.V., Almelo, The Netherlands). BET surface area and porosity measurements were carried out by N_2_ adsorption at 77 K using a Micromeritics 2010 instrument (Malvern Panalytical B.V., Almelo, The Netherlands). Absorption UV–vis spectra were recorded by using a Cary 300 (Agilent Technologies Deutschland GmbH, Waldbronn, Germany) and transformed to a magnitude proportional to the extinction coefficient (K) through the Kubelka–Munk function, F(R∞). In order to estimate band gap energy values of the materials, Tauc plots were used, representing (F(R∞)· hν)n as ordinate and hν as abscissa, where h is the Planck constant, ν is the frequency and n is ½ for indirect semiconductors, as WO_3_ and AgBr are. Morphology of the samples were analyzed by scanning electron microscopy (SEM) using a Hitachi S4800 field emission microscope (Hitachi High-Tech Europe GmbH, Krefeld, Germany) equipped with an EDX detector for chemical analysis. Diffuse reflectance infrared Fourier transform spectroscopy (DRIFTS) was performed on a JASCO FT/IR-6200 IRT-5000 instrument (JASCO Corporation, Tokyo, Japan).

Photocatalytic tests: The photocatalytic behavior of the samples was evaluated in the Rhodamine B (RhB) oxidation reaction under sun-like irradiation. A batch reactor (250 mL) and an Osram Ultra-Vitalux lamp (300 W) (OSRAM GmbH, Munich, Germany) with a sun-like radiation spectrum were used. The intensity of the incident UVA light on the solution was measured with a PMA 2200 UVA photometer (Solar Light Co., Inc., Glenside, PA, USA) and set at 90 W m^−2^ (UVA sensor PMA2110; spectral response 320–400 nm). The catalyst concentration was 1 g L^−1^, and the initial concentration of RhB was 10 ppm. Air was continuously pumped into the solution.

Before each experiment, the photocatalysts were left in suspension with the RhB for 30 min to allow adsorption equilibrium. The evolution of RhB discoloration with illumination time was followed by UV-Vis spectrophotometry (Cary 300).

To test whether photolysis of RhB occurs under the experimental conditions used, an experiment was performed with illumination and no photocatalyst, and no change in the initial concentration of RhB was observed.

## 4. Conclusions

The incorporation of hydrophilic and high-surface-area biochar, produced by pyrolysis of olive pruning waste under CO_2_ atmosphere at 800 °C, into WO_3_/AgBr photocatalyst systems led to an increase in their efficiency when the incorporation of biochar into the photocatalyst was carried out during the photocatalyst synthesis process. Likewise, a percentage of 10 wt.% of biochar in the system gave the best results for the photocatalytic degradation of RhB. The incorporation of BCO significantly increased the surface area and adsorption capacity of the photocatalysts, especially when the biochar was introduced in situ during synthesis. This method resulted in improved particle dispersion and stronger interactions between the biochar and the photocatalytic components, as confirmed by SEM and DRIFT analyses. Overall, the results highlight the potential of agroforestry waste-derived biochar in developing efficient and sustainable photocatalytic materials.

## Figures and Tables

**Table 1:** Proximate analysis of olive branch and ultimate analysis of obtained biochar.

**Proximate Analysis (wt. %)**	
Moisture	7.75
Volatiles	82.3
Ash	4.37
**Ultimate Analysis (wt. %)**	
C	52.69
H	0.66
N	1.23
O	22.10 ^(^*^)^

^(^*^)^ Calculated by difference.

**Table 2:** XRF results for the biochar.

Elements	% (by Weight) *	Elements	% (by Weight) *
Al	0.52	Mg	2.74
Ca	65.43	Na	0.32
Cl	0.24	P	2.32
Cu	0.17	S	0.65
Fe	1.00	Si	2.11
K	24.04	Sr	0.10

* Without contribution of C, H, O and N.

**Table 3:** BET surface area values and band gap values for the different samples.

Sample	S_BET_ (m^2^/g)	Band Gap (eV)
BCO	487	-
AgBr	<1	2.66
WO_3_	20	2.76
WO_3_/AgBr	9	2.65
WO_3_/AgBr MM 1% BCO	11	2.81
WO_3_/AgBr MM 10% BCO	38	2.71
WO_3_/AgBr DP 1% BCO	18	2.79
WO_3_/AgBr DP 10% BCO	37	2.80

**Table 4:** Calculated first-order kinetic constants (k) and determination coefficients (R^2^) for the different samples.

Catalysts	K (min^−1^)	R^2^
AgBr	0.015	0.994
WO_3_	0.010	0.994
WO_3_/AgBr	0.058	0.993
WO_3_/AgBr MM 1% BCO	0.030	0.998
WO_3_/AgBr MM 10% BCO	0.030	0.993
WO_3_/AgBr DP 1% BCO	0.073	0.976
WO_3_/AgBr DP 10% BCO	0.127	0.963

## Data Availability

The raw data supporting the conclusions of this article will be made available by the authors on request.

## Supplementary Materials

The following supporting information can be downloaded at: https://www.mdpi.com/article/10.3390/ijms262110451/s1.
