# Supplementary material for: Enhancing the Photocatalytic Performance of WO3/AgBr Composites Through the Incorporation of Olive Waste-Derived Biochar Obtained Under Controlled Pyrolysis Conditions
Source: Int J Mol Sci. 2025 Oct 28;26(21):10451. doi: 10.3390/ijms262110451 (PMC12607748; doi:10.3390/ijms262110451)
Supplement: Supplementary file 1 [file ijms-26-10451-s001.zip › ijms-3916948-supplementary.pdf]

SUPLEMENTARY INFORMATION

Enhancing the Photocatalytic Performance of WO_3/AgBr Composites through the Incorporation of Olive Waste-Derived Biochar Obtained under Controlled Pyrolysis Conditions

M.C. Hidalgo [1]*, M.D. Alcalá [2], J.A. Navío [1,2], F. Romero-Sarria [1,2]

[1] Instituto de Ciencia de Materiales de Sevilla (ICMS), CSIC - Universidad de Sevilla, Americo Vespucio 49, 41092, Sevilla, Spain
[2] Departamento de Química Inorgánica, Universidad de Sevilla, Profesor García González 1, 41012 Sevilla, Spain
* Correspondence: carmen.hidalgo@csic.es; Tel.: +34 954489630

Recycling experiments were carried out using the WO_3/AgBr DP 10% BCO sample, which showed the highest activity in the degradation of Rhodamine B (RhB). The measurement conditions were the same as in the other experiments: RhB 10 ppm, catalyst concentration = 1 g/L, simulated sunlight (Osram Ultravitalux 300 W) with an intensity of 90 W^{-2} on the surface of the solution (PMA 2200 UVA photometer (Solar Light Co.) and UVA sensor PMA2110; spectral response 320–400 nm). Air was continuously bubbled into the solution. Before each experiment, the photocatalysts were suspended with RhB for 30 minutes to allow adsorption equilibrium. The evolution of RhB discoloration under illumination was monitored by UV-Vis spectrophotometry (Varian Cary 300).

After each cycle, the catalyst was recovered, dried at 90 °C overnight, and reused in the next cycle with a fresh RhB solution (10 ppm). The degradation profiles are shown in Figure S1. The corresponding kinetic constants (k, min^{-1}) were calculated from the linear fit of $\ln(C/C_0)$ versus time during the first 30 minutes of illumination, and the results are presented in Table 4. All fits show good agreement with first-order kinetics ($R^2 > 0.90$). No notable loss of activity is observed during the first four cycles.

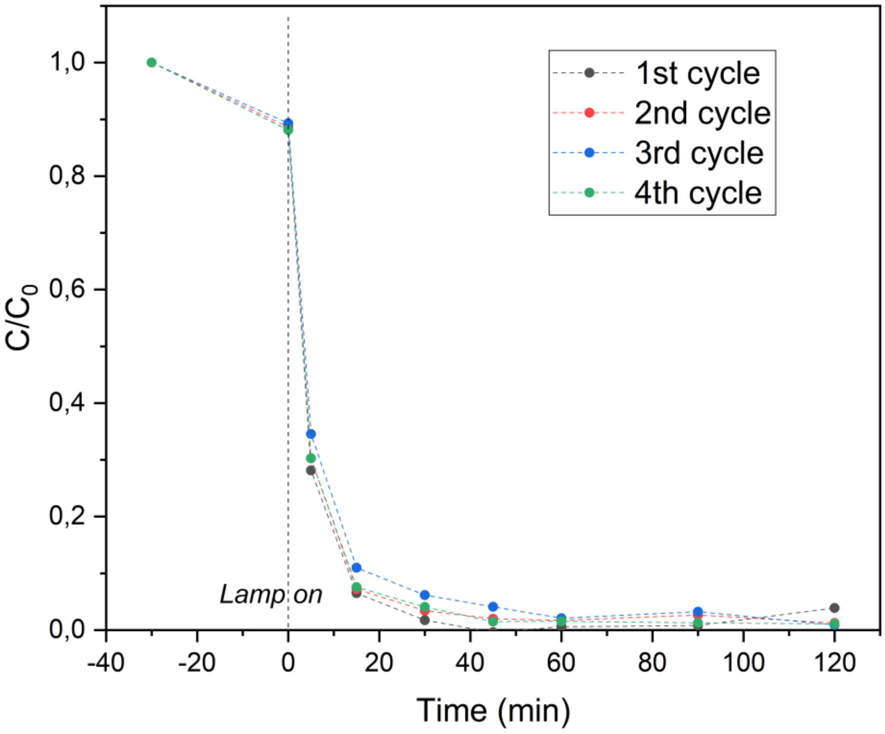

Figure S1. RhB degradation profiles over the sample WO_3/AgBr DP 10% BCO in consecutive cycles.

Table S1: Calculated first-order kinetic constants (k) and determination coefficients (R^2) for the different consecutive cycles over WO3/AgBr DP 10% BCO

WO3/AgBr DP 10% BCO	K(min^{-1})	R^2
1st cycle	0.127	0.963
2nd cycle	0.105	0.913
3rd cycle	0.086	0.910
4th cycle	0.098	0.904
